# The Culture of Herbal Preparations Among Pregnant Women: A Remedy or a Suicide Potion? A Case Report and Mini Review

**DOI:** 10.1155/2020/6186147

**Published:** 2020-03-12

**Authors:** P. P. S. Ossei, A. Appiah-Kubi, F. Ankobea-Kokroe, G. Owusu-Asubonteng, W. G. Ayibor, O. K. Aninkora, J. Taylor, E. A. Fenteng, E. Agyemang-Duah, B. M. Agagli, N. Niako

**Affiliations:** ^1^Department of Pathology, School of Medicine and Dentistry, Kwame Nkrumah University of Science and Technology and Komfo Anokye Teaching Hospital, Kumasi, Ghana; ^2^Department of Obstetrics and Gynecology, University of Health and Allied Sciences, Ho, Ghana; ^3^Department of Obstetrics and Gynecology, School of Medicine and Dentistry, Kwame Nkrumah University of Science and Technology and Komfo Anokye Teaching Hospital, Kumasi, Ghana; ^4^Department of Molecular Medicine, School of Medicine and Dentistry, Kwame Nkrumah University of Science and Technology, Kumasi, Ghana; ^5^Department of Pathology, Komfo Anokye Teaching Hospital, Kumasi, Ghana; ^6^Department of Molecular Biology and Biotechnology, Tezpur University, Assam, India; ^7^Institute of Biochemistry and Molecular Biology, Universität Potsdam, Potsdam, Germany; ^8^Department of Molecular Medicine, Ulm University, Ulm, Baden-Wuerttemberg, Germany

## Abstract

In general, use of herbal remedies and preparations is on the ascendency in recent times among the general population and especially in young pregnant women, and this may be very dangerous due to adverse effects and interactions with drugs. A survey by the World Health Organization revealed that 70–80% of the world population resort to nonconventional medicines especially, herbal medicines in their primary healthcare. A lot of work has been done on the positive effects of herbs on the human body but very few publications on the potential side effects of consuming crude herbal preparations especially among pregnant women or the awareness of the medical team of this problem. Herbal remedies may come with many adverse effects and potentially serious interactions with some conventional medications. However, little is known about the dangers associated with consumption of herbal remedies by pregnant patients. Herbal medicines like their orthodox counterparts act through some mechanisms to bring about their curative effects in the body, and this usually goes out of order when these remedies interact with chemical drugs as a result of a combination of both by the victims. This is a case study to review the use of herbal medicine products among pregnant women, especially adolescent girls for abortive purposes, and also attempts to discuss some of the dangers associated with the use of herbal medicinal products together with conventional drugs during pregnancy.

## 1. Introduction

The use of herbal medicinal products, predominantly used as food supplements, is exponentially increasing [1–3]. It is not uncommon today to wake up to media advertisements promoting different herbal medicinal products. Herbal medicines refer to herbs, herbal preparations, and finished herbal products used in the treatment of disease conditions and for general health maintenance.

Between 1990 and 1997, herbal medicine product usage in the US general population rose by 3.8% [4]. Study has established that consumers of herbal medicine products are mostly female. The general assumption is that when pregnant women use these herbal remedies, they are usually perceived as being natural and hence are free of risks [5, 6]. The sad thing is that many of these herbal preparations are not even certified and many have not passed standardization tests but are used by a growing number of people for preventive and therapeutic purposes. More than 50% of pregnant women take prescribed or nonprescribed medicines at some point in the course of pregnancy [7, 8]. In general, drugs should not be used during pregnancy unless absolutely necessary because many can have teratogenic and abortive effects on the fetus as well as other life-threatening adverse effects on the consuming mother. About 2-3% of all birth defects result from drugs taken to treat a disorder or symptom [9]. However, medicines sometimes are essential for the health of the pregnant woman and the fetus, but the risk and benefits associated with such medicines should be made known to pregnant women by their physicians as these herbal medications and supplements can result in deleterious outcomes for the mother and the fetus [10]. Over the past few years, several eye-catching episodes in some communities indicated adverse effects, sometimes life-threatening, allegedly arising consequential to the ingestion of over-the-counter or crude herbal products or traditional medicines from various ethnic backgrounds [11].

Manufacturers of herbal medicines are largely not regulated and are not required to provide a proof of safety to the Food and Drug Administration (FDA). It is therefore difficult to really monitor the toxic effects of these herbal medicines that are so common these days. These products do not pass scientific tests required for chemical drugs and are not subjected to the approval of the FDA. Most ethnic herbal preparations administered to people do not adhere to dosage or toxicity safety rules but are ingested based on one's discretion, a practice passed down generations and is overwhelmingly common in many communities.

These herbal preparations are mostly made from whole plant parts without selective extraction of the active ingredients but come together with all other compounds [12, 13] that do not break down even after boiling and have very serious physiological implications like poisonous alkaloids and phenolic compounds. Some of them are also extracted using cold extraction with alcohol, another chemical that can adversely impact the liver. Contamination with toxic metals, adulteration, and nonstandardization are a few of the many dangers that usually accompany the preparation of these herbal formulations. Some of these compounds when ingested can interact with anticoagulants and other biological agents to cause spontaneous bleeding, while others can induce abortion when ingested by pregnant women. There is considerable concern about the safety of some herbs, with reports of newborn babies experiencing heart attacks or strokes after the maternal consumption of caulophyllum to induce labor [14]. Some of these side effects are exacerbated when these herbal mixtures interact with pharmaceutical medications, as some patients combine both [15].

Physicians and other health professionals are expected to warn patients against these herbal mixtures and are to ask all patients about these herbal preparations. Dosage taken and number of times taken are all factors that can seriously affect the extent of hazard these preparations pose to an individual since most are not standardized and are therefore not indicated. One sad thing is that most of these victims usually present late at the hospital/clinic after using these herbal products that have already overwhelmed the vital organs; as such, any intervention becomes ineffective due to their already compromised state. Herbal products containing ephedrine have been found to be associated with adverse cardiovascular events, seizures, and more seriously, death [16, 17].

The current research is a case study on herbal medicine use by two pregnant women, one ingesting it as an abortion inducer. It also attempts to discuss some of the dangers associated with the general use of herbal medicinal products as they contain some toxic compounds that have serious health implications. We aim to caution pregnant women, who resort to these remedies, healthcare personnel, and the general populace of the usage of herbal medicinal products, as they are not risk-free.

## 2. Methodology

This study is a case report of two patients who presented with similar symptoms prior to their demise. It was found out that they ingested some crude herbal preparations. The patients were subsequently referred to the Komfo Anokye Teaching Hospital, Kumasi, a 1000-bed hospital located in the middle belt of Ghana that serves the Ashanti, Brong Ahafo, and to some extent the northern part of the country. Patients' data on demographics, clinical summary, and autopsy findings were recorded. Toxicology report on the herbal preparations was also done. The autopsy findings and complications leading to death were based on the criteria of the WHO's International Classification of Diseases version 10 (ICD-10) [18].

The Committee on Human Research Publication and Ethics of the School of Medical Sciences, Kwame Nkrumah University of Science and Technology, and the Komfo Anokye Teaching Hospital, Kumasi, gave approval for the study.

Patients' records were made anonymous throughout the study.

## 3. Case Report 1

### 3.1. Case Summary of the Late C.B. (45 years)

A 45-year-old woman, madam CB, was referred to our emergency unit on 22 July 2018. The patient was referred on account of eclampsia, acute kidney injury, and HELLP syndrome at 33 weeks gestation.

The patient presented there with complaints of epigastric pain and blurred vision of a day's duration.

She was apparently well until a day prior to presentation when she had the above complaints, which became associated with tonic clinic seizures at home with tongue bite; hence, she was rushed to the hospital. At the hospital, she had another episode of tonic clinic seizures. She was stabilized and referred to KATH for further management. The patient was given antihypertensives and anticonvulsant before referral.

At KATH, the blood pressure was high with proteinuria of 3+; however, the SpO_2_ was 99% on room air. She looked very unwell, pale, and not jaundiced with good hydration. Her chest was clinically clear. She had epigastric pain. Symphysis-fundal height of 32 cm was consistent with the gestational age. Lie was longitudinal, and presentation was cephalic. Fetal heartbeat was present and normal. No contractions were felt. On pelvic examination, the vulva and vagina looked healthy. The cervical os was closed, and the cervix was 2 cm long, firm, and posterior. The patient had 500 mL of cola-like urine and bedside clotting of 15 min. Impression of eclampsia with HELLP syndrome with an unfavorable cervix was made. The patient had samples taken for FBC, BUE, creatinine, serum uric acid, and grouping and crossmatch for 4 units of whole blood and 8 units of FFP. She was then prepared for emergency CS after the bedside clotting was corrected with transfusion of 3 FFPs. Immediately postop, the patient was very stable with normal vital parameters. 4 h post-CS, the patient had abdominal distension was tympanic, severally pale, and jaundiced. The uterus was well contracted, and shifting dullness was negative. Her pulse was 116 with blood pressure of 164/112 mmHg.

The patient had made 30 mL of cola-like urine in 4 hours. No bleeding per vaginam was noted. She was continued with the haemotransfusion and the FFP, but unfortunately, her condition did not improve and she ceased breathing. Cardiopulmonary resuscitation was attempted for over 30 min but was unsuccessful; hence, she was declared dead. Her labs came later, and liver indices were all markedly raised.

### 3.2. Postmortem Report

General condition: body of a woman of normal posture. She is 1.60 m in height. She is 45 years old.

Postmortem findings established multiple petechial bleeds in the lungs, liver, and gastric mucosa. There was massive clotted blood collected in the gastrointestinal tract. The postmortem findings are summarised as follows (the cause of death):

(1) Haemorrhagic shock
  - Ingestion of corrosive herbal preparation
(2) Disseminated intravascular coagulopathy

### 3.3. Toxicology Report 1

Test report:

Laboratory no.: GSA LAB No9: 121/FS4/2019

Request: screening for common poisons including herbal poisons

*Description/specimen tested*

Liver—110 g

Stomach and content—320 g

Both kidneys—165 g

Blood sample—10 mL

*Method of analysis*: chemical/chromatography

Sampling procedure: submitted by Central Police, Kumasi, Ashanti

Test results

Liver: common poison/drugs—phenolic compounds detected

Stomach content: common poisons/drugs—phenolic compounds detected

Kidney content: common poisons/drugs—phenolic compounds detected

Blood sample: common poisons/drugs—phenolic compounds detected

Remarks

The samples submitted for analysis tested positive for phenolic compounds. They are basic chemical compounds of plant origin, which have pharmacological effects on humans and animals. They are corrosive/toxic and harmful depending on the amount ingested.

## 4. Case Report 2

### 4.1. Case Summary of the Late SM (19 years)

A 19-year-old lady, SM, was rushed to the emergency department of Frimpong-Boateng Medical Center on 25/03/2019 with complaints of severe abdominal pains maximal in the epigastric region, abdominal cramps, and dizziness.

She was unwell. She was afebrile, jaundiced, and pale and had severe abdominal tenderness. She had apparently ingested a brownish-red herbal product to abort a pregnancy. She also took Alusil Plus Syrup when the abdominal pains started. The patient died shortly on arrival while being stabilized at the emergency department.

### 4.2. Postmortem Report

General condition: body of a young woman of normal posture. She is 1.65 m in height. She is 19 years old.

Postmortem examination established multiple petechial bleeds in the lungs, liver, and gastric mucosa. There was massive gastrointestinal bleed (about 1.6 L) and mucosal corrosion. The postmortem findings are summarised as follows (cause of death):

(1) Haemorrhagic shock
  - Massive gastrointestinal bleed
  - Ingestion of corrosive herbal product
(2) Disseminated intravascular coagulopathy

### 4.3. Toxicology Report

Test report:

Laboratory no.: GSA LAB No9: 128/FS6/2019

Request: screening for common poisons including herbal poisons

*Description/specimen tested*

Liver—89 g

Stomach—324 g

Both kidneys—155 g

Blood sample—10 mL

*Method of analysis*: chemical/chromatography

Sampling procedure: submitted by Nkawie Police, Ashanti

Test results

Liver: common poison/drugs—phenolic compounds detected

Stomach content: common poisons/drugs—phenolic compounds detected

Kidney content: common poisons/drugs—phenolic compounds detected

Blood sample: common poisons/drugs—phenolic compounds detected

Remarks

The samples submitted for analysis tested positive for phenolic compounds. They are basic chemical compounds of plant origin, which have pharmacological effects on humans and animals. They are corrosive/toxic and harmful depending on the amount ingested.

In a related case, Lab. No. 20/FS5/2019, the herbal concoctions tested positive for phenolic compounds. The Alusil Plus Syrup contained hydroxide, magnesium hydroxide, and magnesium trisilicate, and the tablet tested positive for ciprofloxacin.

## 5. Discussion

The laws of the land frown on abortions of any form in the hospitals except when there is a strong medical indication to this effect. Religious beliefs and almost every community in the Ghanaian setting also frown upon abortions of any form especially among young reproductive girls who usually resort to herbal concoctions and ignorantly combine these with orthodox medicines. The use of herbal remedies by pregnant women is very common these days either for medicinal use or for abortive purposes. Young girls who for the fear of being school dropouts are forced to resort to crude herbal concoctions to avoid the ignominy and disgrace of being school dropouts.

In the first case was a woman who from the clinical summary and the pathological report, coupled with the toxicology report, had ingested a herbal preparation not for the purpose of abortion but experienced serious complications leading to her demise just as in the second case who also ingested the herbal preparation to induce abortion of the fetus. The findings from both cases overlap as seen from the postmortem findings and similar toxicology report.

Ingesting crude herbal preparation during pregnancy not only has teratogenic effects but also has serious life-threatening complications for the mother. Although quite a number of them are safe, some herbal medicines come with risks. Contamination with heavy metals, doping with Western medicines, and inclusion of prohibited ingredients are very common in ethnic herbal medicines. Some of these herbs are hepato- or nephrotoxic (as was the case for the first victim), and some interact with chemical drugs or orthodox medicines to cause adverse conditions like intravascular coagulopathy and even death [19]. Herbalists over the years have recommended some herbs as abortifacient [20] although no evidence really supports this claim as the adverse effects of these herbs far outweigh their efficacy to produce desired positive results or remedy.

**Figure 1:**
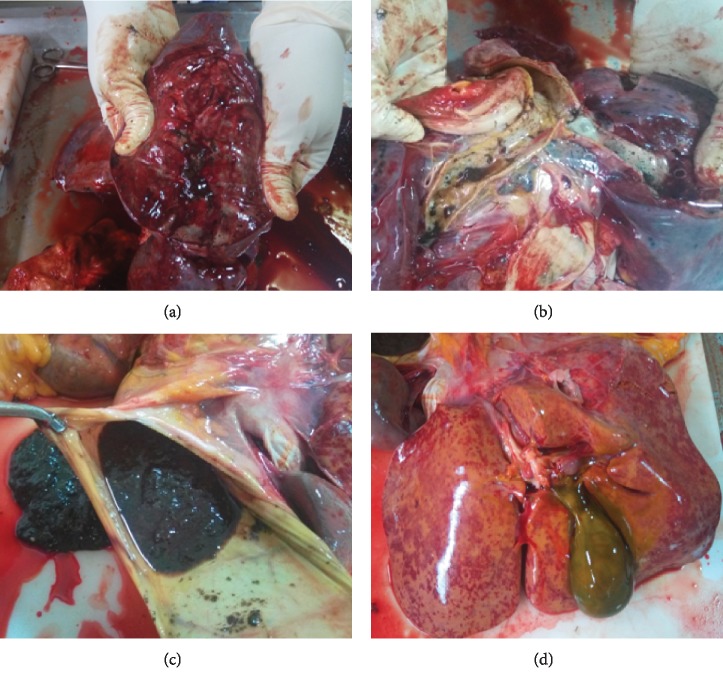
Postmortem findings. (a) Lung with multiple petechiae; it is congested, consolidated, and markedly edematous. (b) Trachea filled with semiformed and undigested herbal preparation and erosion of the intima of the trachea. (c) Gastric cavity showing a massive herbal preparation inducing an extensive gastritis. There is hyperaemia and erosion of the gastric mucosa; the wall is thin and distended. (d) Liver with extensive and diffuse petechial bleeds. Gall bladder markedly congested and extended.

**Figure 2:**
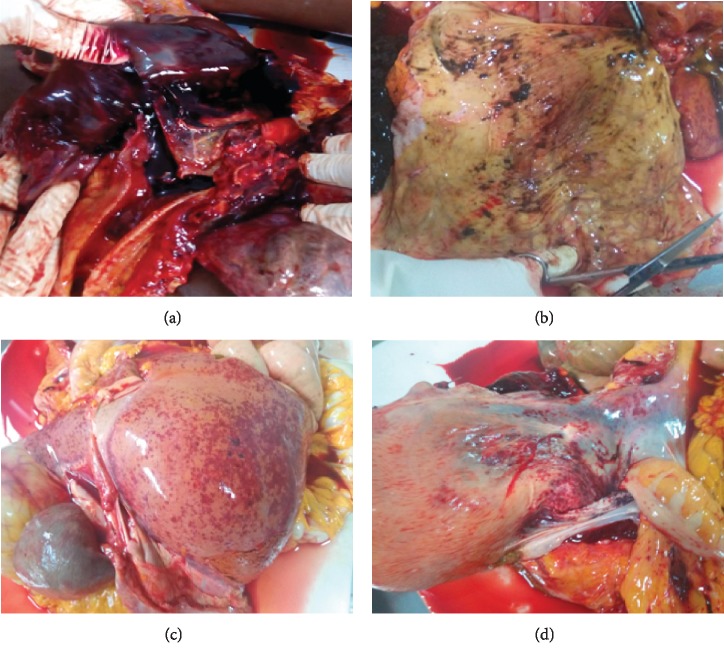
Postmortem findings. (a) Trachea showing massive bleed from congested lung parenchyma and pleural petechial bleed. (b) Stomach wall with completely effaced gastric mucosa and intramucosal bleed. (c) Enlarged liver with extensive DIC. (d) Diaphragmatic region showing extensive petechiae.

Multiple petechial bleeds in the liver and lungs result from injuries to these organs (Figure 1(d)). There is also erosion to the gastric mucosa as a result of the corrosive nature of the compounds contained in these concoctions (Figure 1(b)), and if appropriate and immediate medical attention is not sought, the victim dies from multiple failure, chiefly among them being the liver, which is totally overwhelmed as seen in these cases (Figures 1(d) and 2(c)). Phenolic compounds and other phytochemicals contained in these remedies are extremely toxic as no exclusive extraction protocol is followed and become worse when there is interaction with chemical drugs as seen in the second case. It became apparent that the victim combined the herbal powder with an orthodox chemical drug (Alusil Plus, which contains aluminum silicate). Evidence for the interaction between active ingredients in herbal preparations and chemical drugs has well been documented [21]. The phytochemicals contained in these crude herbal preparations/remedies have the potential to modulate cytochrome P450 activity and thus participate in interactions with conventional drugs, which are usually life-threatening. Herbal preparations from plants such as milk thistle, ginseng, garlic preparations, Angelica dahurica, and liquorice have all been found to have this potential [22].

In both cases, findings from postmortem examination established disseminated intravascular coagulopathy (Figures 1 and 2), probably as a result of interaction of the phytochemicals with an orthodox chemical drug or with blood clotting factors. Herbal medicines have been traditionally used in the management of cardiovascular disorders and can play a role in platelet function and have the potential of altering platelet function tests, as well as some coagulation parameters. Herbal medicines, such as the ones containing St John's wort, ginger, ginseng, garlic, motherwort, and willow bark, have been found to affect platelet aggregation [23].

In the first case, the patient presented with complaints of epigastric pain and blurred vision of a day's duration. Interaction of active ingredient in herbal medicines with drugs has been found to compromise vision [24], and there are lots of documented literature on patients with gastric pains from the toxic and corrosive effects of crude and unstandardized herbal preparations. The reports from postmortem examination confirmed the corrosive effect of the ingested herbal preparation on the gastric wall (Figures 1(c) and 2(b)) culminating in gastric bleeding among other varied effects on other organs, especially the liver which is tasked with neutralizing these toxins in the drug. This and other adverse effects associated with herbal medicines may result from contamination of these products with toxic metals, adulteration, misidentification, or substitution of herbal ingredients as well as improperly processed or prepared products [25].

## 6. Conclusion

Thus, increased knowledge of herbal medicines/herbal medicine-drug interactions would enable healthcare providers and patients (pregnant women) to be alert to the potential of interactions. This would also help to reduce the risk associated with any drug intake. A future consideration of adjusting abortion laws should be prioritized since majority of young women take these herbal preparations as abortion inducers. Finally, herbal medicines should be appropriately and comprehensive labeled, including the potential for drug interactions.

## Abbreviations

KATH: Komfo Anokye Teaching Hospital
FDA: Food and Drug Administration
GSA: Ghana Standards Authority
DIC: Disseminated intravascular coagulopathy.
